# Landscape ecological concepts in planning: review of recent developments

**DOI:** 10.1007/s10980-021-01193-y

**Published:** 2021-01-28

**Authors:** Anna M. Hersperger, Simona R. Grădinaru, Ana Beatriz Pierri Daunt, Carole S. Imhof, Peilei Fan

**Affiliations:** 1 Head of Land Use Systems Group, Land Change Science Research Unit, Swiss Federal Research Institute WSL, Zürcherstrasse 111, 8903 Birmensdorf, Switzerland; 2 Centre for Environmental Research and Impact Studies, University of Bucharest, Bucharest, Romania; 3 Land Change Science Research Unit, Swiss Federal Research Institute WSL, Zurich, Switzerland; 4 School of Planning, Design, and Construction and Center for Global Change and Earth Observations, Michigan State University, Michigan, USA

**Keywords:** Landscape ecology, Bibliographic analysis, Socio-ecological systems, Resilience, Landscape services, Ecosystem services, Green infrastructure, Multifunctionality, Landscape perception, Sense of place

## Abstract

**Context:**

Landscape ecology as an interdisciplinary science has great potential to inform landscape planning, an integrated, collaborative practice on a regional scale. It is commonly assumed that landscape ecological concepts play a key role in this quest.

**Objectives:**

The aim of the paper is to identify landscape ecological concepts that are currently receiving attention in the scientific literature, analyze the prevalence of these concepts and understand how these concepts can inform the steps of the planning processes, from goal establishment to monitoring.

**Methods:**

We analyzed all empirical and overview papers that have been published in four key academic journals in the field of landscape ecology and landscape planning in the years 2015–2019 (n = 1918). Title, abstract and keywords of all papers were read in order to identify landscape ecological concepts. A keyword search was applied to identify the use of these and previously mentioned concepts in common steps of the planning cycle.

**Results:**

The concepts *Structure*, *Function*, *Change*, *Scale*, *Landscape as human experience*, *Land use*, *Landscape and ecosystem services*, *Green infrastructure*, and *Landscape resilience* were prominently represented in the analyzed literature. Landscape ecological concepts were most often mentioned in context of the landscape analysis steps and least in context of goal establishment and monitoring.

**Conclusions:**

The current literature spots landscape ecological concepts with great potential to support landscape planning. However, future studies need to address directly how these concepts can inform all steps in the planning process.

**Supplementary Information:**

The online version of this article (10.1007/s10980-021-01193-y) contains supplementary material, which is available to authorized users.

## Introduction

Prompted by fast and extensive landscape changes throughout the world, landscape ecology aims to provide policy relevant information about landscape change and form the base for landscape management, design and policy (Wu 2013; Mayer et al. 2016). The discipline has a long tradition in reaching out and building bridges to fields of action such as landscape sustainability (Wu 2010), landscape approach (Reed et al. 2016), landscape design (Nassauer and Opdam 2008) and regional and landscape planning (Forman 2008). The contribution of landscape ecology to inform planning and research management has been addressed in conceptual and empirical studies (see e.g., Ahern 1999; Pedroli et al. 2006; Opdam et al. 2013; Wu 2013; Milovanović et al. 2020). Few studies have also analyzed how landscape ecology has been used in landscape planning practices and plan making (e.g., Termorshuizen et al. 2007; Bjärstig et al. 2018; Trammell et al. 2018).

How landscape ecology has reached out to landscape planning, i.e., the focus of this research, is especially interesting. Landscape ecology is an interdisciplinary scientific discipline that focuses on spatial pattern and heterogeneity, and specifically their characterization and description over time, their causes and consequences and how humans manage those (Turner et al. 2001). The conceptual and theoretical core of landscape ecology links natural and social sciences to understand landscapes as arenas where structural features and social construction converge (Pinto-Correia and Kristensen 2013).

Landscape planning is prominent across the world as an integrated, collaborative practice on a regional scale (Steiner 2008; Selman 2012) and benefits from landscape ecology in manifold ways. It focuses often on rural areas or open landscapes, where conflicts between urban sprawl and recreational landscape values, agricultural production and nature conservation, and renewable energy production and aesthetics dominate (Mann et al. 2018). Landscape planning greatly varies from place to place and can be integrated into the institutions (e.g., in Germany), provide an input into strategic spatial planning (e.g., in Switzerland), be conducted as an ad hoc initiative (e.g., in the USA) or be largely missing (e.g., in Romania) (Hersperger et al. 2020).

Landscape planning as an academic field is undertheorized, as evidenced by the fact that very few scientific journals are devoted to landscape planning (with the notable exception of “Landscape and Urban Planning”). However, landscape planning has a strong tradition in addressing procedural aspects that has led to established planning procedures. They operationalize the planning process through a sequence of steps and are well suited to investigate the link between landscape ecology and planning. Well-known examples are Steiner’s Ecological Planning Model (Steiner 2008), Steinitz’ Framework for Landscape Planning (Steinitz 2012), and Ahern’s Framework Method for Sustainable Ecological Planning (Ahern 1999). In this line of work are also proposals that explicitly address landscape ecological planning (Wang et al. 2001; Hersperger 2006; Miklós and Špinerová 2019). The pragmatic conceptualization of the planning process into a sequence of steps should not undermine the fact that landscape planning, like any kind of spatial planning, must be accepted as an ongoing political activity that is geared towards negotiation and conflict resolution between different public and private actors, within an arena of dynamic multi-level power relations and funding regimes (Oliveira and Hersperger 2019).

Landscape ecological concepts hold a great potential for integrating landscape ecological knowledge into landscape planning (Botequillha Leitao and Ahern 2002). We understand “concept” in line with Merriam-Webster's online dictionary as representing an abstract or generic idea generalized from particular instances (Merriam-Webster 2020). In the case of landscape ecology, these ideas can refer to the representation and organization of landscape elements (e.g., in terms of connectivity), to landscape characteristics (e.g., patterns) or to frameworks for landscape analysis (e.g., landscape services). Most of these concepts have an intrinsic spatial nature. The goal of this paper is to review recent publications to assess the use of landscape ecological concepts in planning. Specifically, we address the following research questions:

1. Landscape ecological concepts: What are they? How frequently are they mentioned in current research?
2. How have landscape ecological concepts been integrated into landscape planning?

We present results on the identified landscape ecological concepts, their prevalence and integration into planning. The discussion centers on the use of landscape ecological concepts and on promising opportunities for landscape ecological concepts in planning.

## Methods

### Data collection

To collect our data, we adopted the PRISMA approach for systematic review (Moher et al. 2009). Four key journals in the field of landscape ecology were selected to conduct the analysis, respectively Landscape Ecology (LE), Landscape Online (LO), Current Landscape Ecology Reports (CLER), and Landscape and Urban Planning (LUP). The choice was based on (1) the relevance for landscape ecology science and (2) the clear linkages between landscape science into planning, based on aim and scope descriptions (for details see Supplementary material 1). All articles published in the four journals in the period 2015–2019 were downloaded and served as a basis for the analysis (n = 1918). The five years period was considered long enough to prevent distortions caused by special issues and short enough to keep the workload manageable.

### Identification and prevalence of landscape ecological concepts

Since we are not aware of a list of well-accepted landscape ecological concepts that would be suitable for our analysis, we resorted to an early publication that identified landscape ecological concepts when discussing landscape ecology and its potential application to planning (Hersperger (1994). To account for recent developments, we analyzed the sample of publications described above. Based on reading the title, abstract and keywords of all papers, an extensive list of concepts, topics and types of landscapes was extracted (n = 39). The high number can be explained by the fact that these concepts are often rather specific because their names have been taken directly from the paper. Each concept was assigned to a type (landscape ecology sensu stricto, ecology, land change science, planning/management, landscape perception). These types were used for a first grouping. We distinguished concepts from (1) topics, in the sense that the later are considered a theme addressed within the broader scientific discourse rather than abstract or generic idea in landscape ecology (e.g., climate change, sustainability), and (2) types of landscapes (e.g., agricultural landscapes, historic landscapes). The extensive list of concepts extracted from the first screening went through subsequent regrouping. Synthesizing led to the definition of seven additional concepts, where the detailed entries in the original list are often used to describe the concepts.

Then, all 1918 papers went through a keyword search to identify the use of early and additional concepts. We used the “pdfsearch” package in R programming language, version 3.6 (R Core Team 2020; LeBeau 2018) and searched for singular and plural forms and different variations of the concepts, e.g., for “holism”, we also searched for “holistic”; and for “classification of landscape types”, we searched for “classification of landscape”, “landscape classification”, “landscape classes” (see Supplementary material 1, Table A). Results are reported as frequency of use per journal and/or period and can be interpreted as an indicator of how prevalent these concepts are.

### Integration of landscape ecological concepts into planning

The title, abstract and keywords of the papers (n = 1918 articles) were screened to identify papers which might show how landscape ecological concepts are integrated into planning. A subsample of n = 131 papers was identified, which was further assessed for eligibility by full-reading. We retained 84 papers: 52 empirical papers and 32 overview papers for further analysis (see Supplementary material 4). The overview papers were further differentiated into reviews of scientific papers, evaluations of plans and projects, and frameworks and essays.

Full reading of the empirical papers allowed us to evaluate how landscape ecology concepts have been integrated into each planning step of the planning cycle. The planning steps were derived from works by Steiner (2008), Steinitz (2012), and Botequillha Leitao and Ahern (2002) (see Table 1). To systematically collect the data, we used a protocol which addressed the following questions: (a) which type of planning is addressed by the paper?, (b) to which planning level does the paper refer to?, (c) which concepts are integrated in any of the planning steps described above? The insights from the overview papers on the integration of landscape ecological concepts into planning were synthesized after careful reading. To ensure systematic interpretation, all readers applied the protocol in two articles, and we calibrated the assessments and interpretation through detailed discussions (for more detail see Supplementary material 2).

**Table 1:** Steps of the planning process for the analysis, derived from Steiner (2008), Steinitz (2012) and Botequillha Leitao and Ahern (2002)

Steiner (2008)	Steinitz (2012)	Botequillha Leitao and Ahern (2002)	Steps of the planning process used in this study
Goal establishment	Is the current study area working well? (evaluation model)	Diagnosis	**Goal establishment** What are the problems? What should be achieved?
Inventory and analysis of biophysical and socioeconomic processes (different scales, regional to local)	How should the study area be described? (representation model) How does the study area work? (process model)	Focus Analysis; and public participation	**Analysis** Biophysical and socioeconomic processes: description and assessment
Concepts and options	How might the study area be altered? (change model) (alternative futures) What differences might the changes cause? (impact model)	Prognosis: alternative plans and evaluation, public participation	**Alternative options** How might the landscape be altered? Impact of the different options?
Plan (chosen option)	How should the study area be changed? (decision model)	Synthesis	**Preferred plan** Suggested actions
Education and participation (8)	–(stakeholder input)	In Analysis and prognosis, explicitly	**Participation and Communication** Throughout the planning process
Detailed designs for the chosen option	–	–	–
Implementation	–	Implementation	–
Administration and monitoring	–	Monitoring	**Monitoring**

## Results

### Landscape ecological concepts in current research

Table 2a lists the eight concepts discussed by Hersperger (1994). GIS was also mentioned as a concept but was omitted from our analysis since it has developed into a widely used tool. Over time, many differentiations within the composite concept of *Structure, function, change* have been developed. The three components of the concept now form the basis of many quantitative landscape assessments, e.g., with landscape metrics (Costanza and Terando 2019), and change (Land change) became a science of its own. Thus, *Structure*, *Function* and *Change* will be treated as separate concepts in the quantitative analysis.

Our analysis of the papers published in the past 5 years identified seven additional concepts (Table 2b). In the following paragraphs, the concepts are described, while the potential of the concepts for linking landscape ecology and planning will be explored in the discussion section.

#### Landscapes as socio-ecological systems

Socio-ecological systems, also called coupled human–environment (H-E) systems, provide a useful integrated analytical framework to understand the relationships between humans and environment (Holling 2001; Miyasaka et al. 2017). While heterogeneity, hierarchy, and feedback mechanisms are essential characteristics of socio-ecological systems, different integrated approaches have been developed to understand socio-ecological systems, including system dynamic models, spatial optimization models, spatial Bayesian Network models, and agent-based models (Liu et al. 2007; Le et al. 2012; Miyasaka et al. 2017).

#### Landscape resilience

Holling introduced the concept of resilience in ecological systems in 1973, as the persistence of relationships within a system that measures the ability of these systems to absorb changes (Holling 1973). Specifically, *Landscape resilience* is the capacity of a landscape/system to maintain the landscape process, ecological, economic, and social functions under changing conditions, and under diverse physical and socioeconomic challenges (Beller et al. 2018; Mock and Salvemini 2018). Schippers et al. (2015) suggest that resilient landscapes are determined by landscape diversity and spatial organization, and that greater variation in ecosystem elements provides more ecosystem services and enhances the resilience of landscape.

#### Landscape and ecosystem services

The Millennium Ecosystem Assessment (MEA) (2005) popularized the ecosystem services concept in the early 2000s. The mapping and assessment of ecosystem services have since been high on the agenda of many administrations. Like ecosystems, landscapes provide vital services to people (Keller and Backhaus 2020), i.e., the many and varied benefits to humans gifted by the natural environment. The ecosystem services concept is by far more prevalent in the scientific discourse than the landscape service concept. Some of the ideas that have inspired the development of the landscape service concept have been taken up by the broadening ecosystem services concept, as witnessed by the formulation “ecosystem services in the landscape context” and by the landscape approach. Termorshuizen and Opdam (2009) point out that in the context of landscape and ecosystem service discussions, “landscape” is used for all kinds of areas, whereas “ecosystem” is often associated with protected areas and biodiversity.

#### Green infrastructure

The concept of *Green infrastructure* refers to the network of green and blue elements such as remnant native vegetation, parks, private gardens, golf courses, street trees, and engineered options such as green roofs, green walls, bio filters, and rain gardens (Norton et al. 2015). Green infrastructure can promote ecosystem and human health in urban areas (Tzoulas et al. 2007). Unlike other types of public infrastructure such as roads, storm water systems, and schools, green infrastructure is often considered as amenity, not as a necessity (Benedict et al. 2006). Furthermore, the contribution of green infrastructure to mitigating high temperatures in urban landscapes, and to adapt to climate change more generally, has been widely recognized (Norton et al. 2015).

#### Multifunctionality

The concept of *Multifunctionality* highlights that landscapes tend to have multiple outputs and provides perspectives for “delivering joined-up policy where its core property of interactivity can be harnessed in ways that produce qualities valued by people” (Selman 2009). The concept developed from a feature of European agricultural landscapes (Otte et al. 2007) into an interdisciplinary concept which allows for understanding and analyzing landscapes from various perspectives, e.g., social, cultural, ecological, aesthetic (Bolliger et al. 2011). Landscapes serve multiple functions at the same time through (1) the same piece of land serving several uses, (2) an area being made up by many small areas dedicated to specific uses, and (3) interactions of uses (Otte et al. 2007). The concept is in line with the current shift from taming nature to reconnecting with nature, reflected by research directions on human-nature interactions, such as socio-ecological systems and human-wildlife coexistence (König et al. 2020).

#### Land use

Land use can be defined as “the total of arrangements, activities and inputs undertaken in a certain land cover type to produce, change or maintain it” (FAO 1997; Verburg et al. 2015). In other words, land use indicates the way geographic space is occupied by society and its activities. Typical land use categories include agriculture, grazing, forestry, transportation, residential, commercial, and recreation. The type of management and the intensity of land use affect stress and potential environmental degradation. The concept allows an integrated focus on structural and functional landscape aspects while addressing human agency.

#### Landscape as human experience

The concept of *Landscape as human experience* evolved from early conceptual research on perceptual and psychological processes related to nature, such as the framework by Kaplan (1995) on human-nature relationships and the conceptual model by Gobster et al. (2007) on the relationship between aesthetics and ecology. The concept flourished with the application of new technologies that allowed for quantitative measurements of human experience, such as stress measurement based on salivary cortisol (Ward Thompson et al. 2012). The concept integrates social and cultural processes affecting landscape valuation and includes, among others, aspects of sense of place and soundscapes. Sense of place is particularly used to reflect the way people or communities attribute meaning, value, and significance to landscapes (Soini et al. 2012). The term soundscape is most often used to refer to the acoustic environment as perceived, experienced and/or understood by individuals and communities (Alleta et al. 2016).

#### Prevalence of landscape ecological concepts

**Fig. 1:**
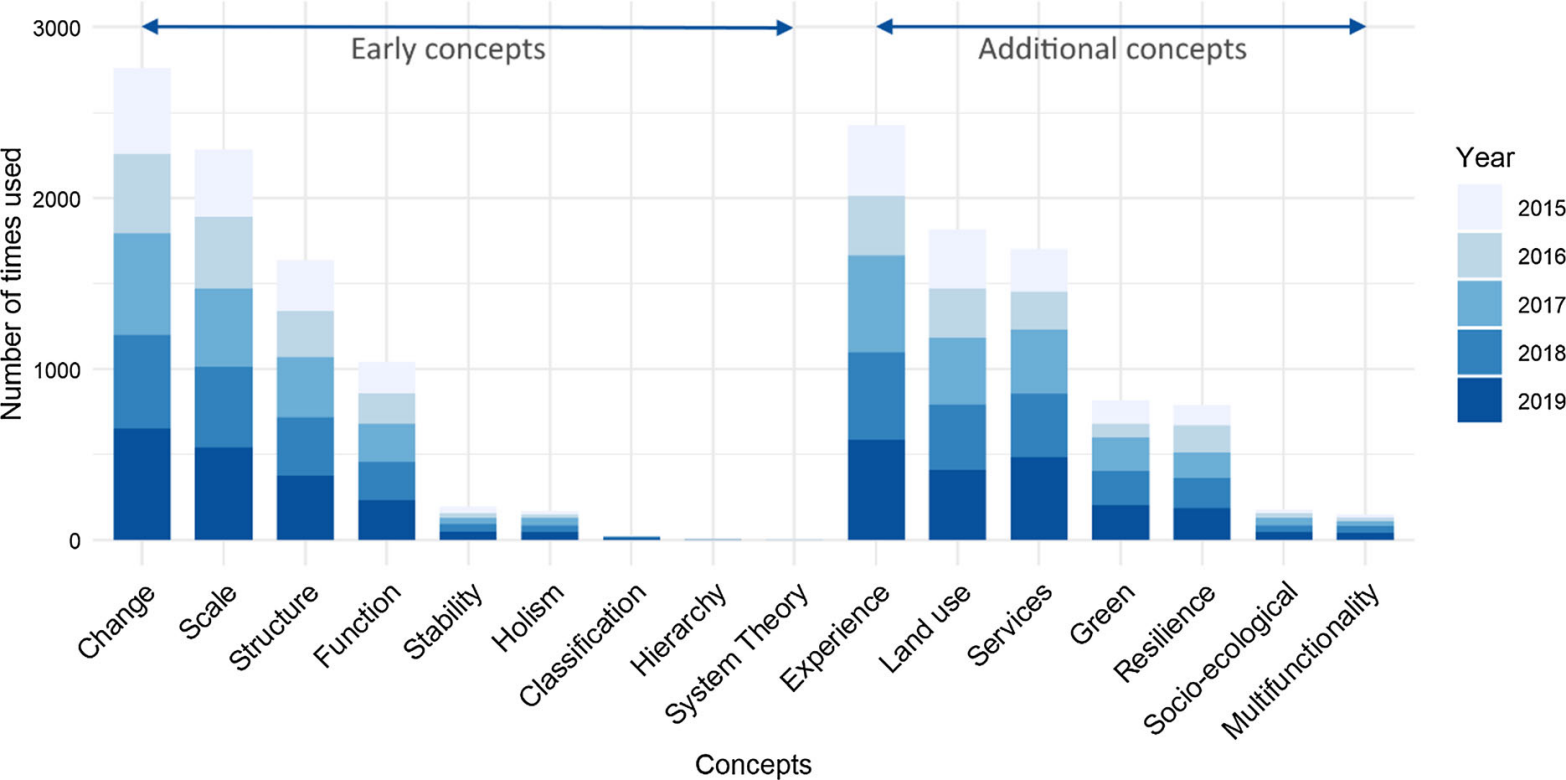
Number of times a concept was used in the 1918 papers published in the years 2015–2019 by the journals Landscape Ecology, Landscape Online, Current Landscape Ecology Reports, and Landscape and Urban Planning. Early concepts are listed on the left, additional concepts on the right. For the full name of concepts, see Table 2. The concepts *Change*, *Scale*, *Structure*, *Function*, *Landscape as human experience*, *Land use*, *Landscape and ecosystem services*, *Green infrastructur*e and *Resilience* were mentioned more than 500 times

Findings of the keyword search show that four of the early concepts in Table 2a are frequently used in today`s publications, namely *Structure*, *Function*, *Change* and *Scale* (Fig. 1). Concepts that refer to theories are rarely mentioned in our sample, i.e., *Hierarchy theory* (12 mentions), *General system theory* (two mentions), and *Chaos theory* (no mentions). Findings further show that three of the additional concepts in Table 1b are widely used in today`s publications: *Landscape as human experience*, *Land use* and *Landscape and ecosystem services* (Fig. 1). They are followed by *Green infrastructure* and *Resilience*. *Socio-ecological systems* and *Multifunctionality* are rarely mentioned. The numbers per year remained rather stable (Fig. 1).

**Table 2:** Landscape ecological concepts. Table 2a Early concepts (description and references based on Hersperger 1994); Table 2b Additional concepts that were derived from papers published in 2015-2019 in the journals Landscape Ecology, Landscape Online, Current Landscape Ecology Reports, and Landscape and Urban Planning

	Description	Abbreviation in the figures
**Table 2a: Early concepts**		
Structure, function, change	Scientific framework of landscape ecology based on the following three characteristics of the landscape system: *structure*: spatial relationship between patches, corridors and the matrix; *function*: determined by the ecological processes, as the flow of energy, material, animals and plants across the landscape; *change*: product of interaction of structure and function over time (Forman and Godron 1986)	Structure Function Change
Stability	(a) Landscapes are considered metastable, a state of being in equilibrium, but susceptible to being diverted to another equilibrium; (b) stochastic view (Forman and Godron 1986; Botkin 1990)	Stability
Chaos Theory	A way to explain system behavior where, despite rules, systems can be fundamentally unpredictable and behavior is sensitive to initial conditions; it expands the traditional understanding of changes in physical and social systems (Cartwright 1991)	Chaos
Scale	The concept of scales allows analyses at different levels of a hierarchical system, whereas landscape might appear to be heterogeneous at one scale but quite homogeneous at another scale (Forman 1987; Meetenmeyer and Box 1987	Scale
Hierarchy Theory	Hierarchy theory developed as a framework to analyze systems of a certain type of complexity. A hierarchy-theory approach towards landscape ecology recognizes that landscape ecology extends over many spatial and temporal scales (Allan and Starr 1982; Urban et al. 1987)	Hierarchy
General Systems Theory	General systems theory formalizes the way a system, such as a landscape, is perceived. It stresses the hierarchical order of nature as an open system and cross-linkages between various components (Naveh and Lieberman 1984)	GSD
Holism	The basic concept of holism is that holistic entities have an existence other than the mere sum of their parts, and that reality consists of wholes in a hierarchical structure (Smuts 1926; Zonneveld 1990)	Holism
Classification of landscape types	The classification of landscapes is based on a description of landscape attributes, such as structural characteristics or land-use units (Zonneveld 1990)	Classification
**Table 2b: Additional concepts**		
Landscapes as socio-ecological systems	An integrated analytical framework to understand the relationships between humans and the environment, stressing a systems perspective on landscapes and the integration of humans and nature (Holling 2001; Miyasaka et al. 2017)	Socio-ecological
Landscape resilience	The capacity of a landscape to maintain landscape processes as well as ecological, economic, and social functions under changing conditions, and under diverse biophysical and socioeconomic challenges (Beller et al. 2018; Mock and Salvemini 2018)	Resilience
Landscape and ecosystem services	An assessment framework for services provided by landscapes and demanded by humans (Keller and Backhaus 2020)	Services
Green infrastructure	A strategically planned network of natural and semi-natural areas, designed and managed (Norton et al. 2015)	Green
Multifunctionality	Within a landscape, the same piece of land can serve several uses while an area can contain many small areas dedicated to specific uses and host interactions of uses (Otte et al. 2007)	Multifunctionality
Land use	The management and modification of the landscape that reflect intentional human imprints (FAO 1997; Verburg et al. 2015)	Land use
Landscape as human experience	Landscapes as perceived by humans often serves as a starting point for action, including examples of visual landscape, soundscape, sense of place (Gobster et al. 2007; Soini et al. 2012; Aletta et al. 2016)	Experience

**Fig. 2:**
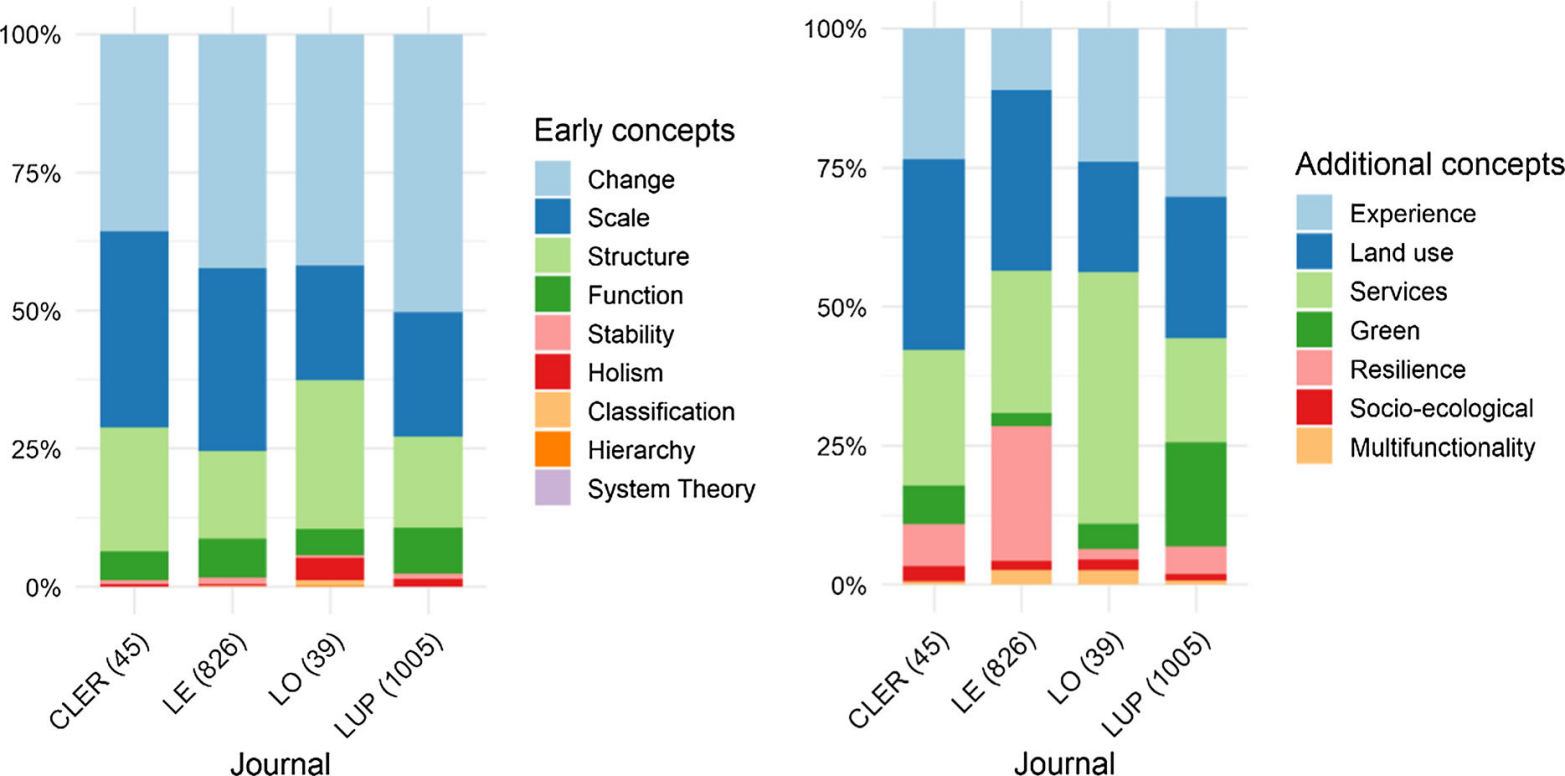
Share in the use of each concept by the journals Current Landscape Ecology Reports (CLER), Landscape Ecology (LE), Landscape Online (LO), and Landscape and Urban Planning (LUP) in the 1918 papers published in the years 2015–2019. Numbers in brackets after journal abbreviations refer to the number of publications in the five years. Left (Table 2**a** refers to early concepts; Right (Table 2**b**) to additional concepts. For the full name of concepts, see Table 2. Journals clearly differ in terms of the prevalence of landscape ecological concepts

Journals clearly differ in terms of the prevalence of landscape ecological concepts. Regarding early concepts, *Change* has been the most prominent concept in all four journals, followed by *Scale* and *Structure* (Fig. 2a). In Landscape and Urban Planning (LUP) *Change* is relatively prominent, in Landscape Online (LO) *Structure*, and in Landscape Ecology (LE) and Current Landscape Ecology Reports (CLER) *Scale* (Fig. 2a, Table B in Supplementary material 3). The analysis of the additional concepts shows that certain concepts are more prominent in certain journals. For example, papers referring to *Landscape resilience* are predominantly published in Landscape Ecology (LE), while articles addressing *Landscape and ecosystem services* are most prominent in the journal Landscape Online (LO) (Fig. 2b, Table C in Supplementary material 3).

**Fig. 3:**
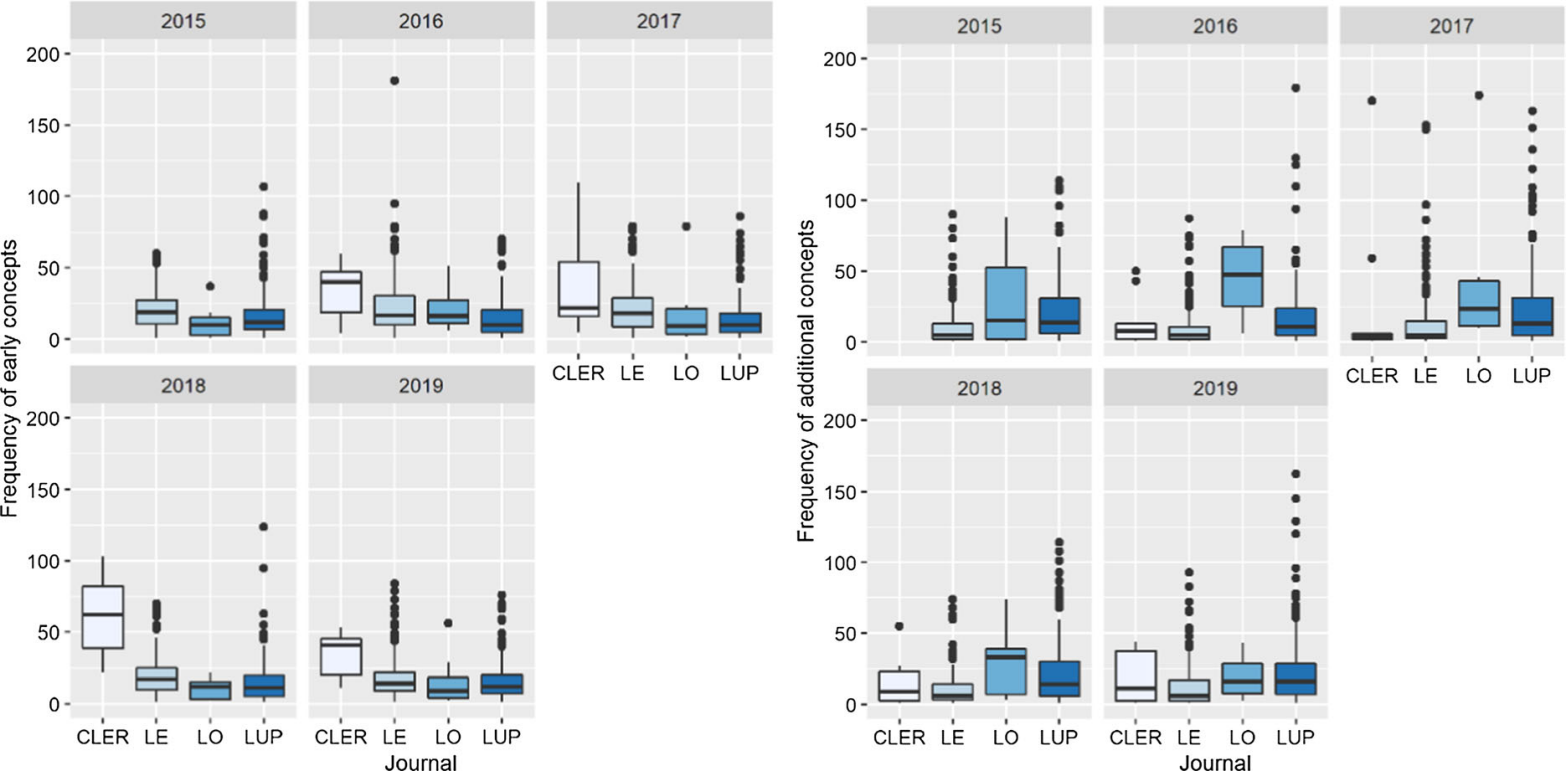
Average number of times a concept was used in a single publication by the journals Current Landscape Ecology Reports (CLER), Landscape Ecology (LE), Landscape Online (LO), and Landscape and Urban Planning (LUP) in the 1918 papers published in the years 2015–2019. Left (Table 3**a**) refers to early concepts; Right (Table 3**b**) to additional concepts. The journals Landscape and Urban Planning (LUP) and Landscape Ecology (LE) regularly publish articles that clearly focus on certain concepts, i.e., a concept is used more than 100 times per article (a and b)

The journals Landscape and Urban Planning (LUP) and Landscape Ecology (LE) regularly publish articles that clearly focus on certain concepts, i.e., a concept is used more than 100 times per article (Fig. 3a and b). Articles published in Current Landscape Ecology Reports (CLER) use early concepts more frequently than articles published in any of the other three journals (Fig. 3a). Furthermore, the concept *Holism* is most often present in papers published by Landscape Online (LO). Interestingly, we found that in the journals Landscape Online (LO) and Landscape and Urban Planning (LUP) the additional concepts are more prevalent than the early concepts, whereas in Current Landscape Ecology Reports (CLER) and Landscape Ecology (LE) we see the inverse pattern (Fig. 3a, b).

#### Integration of landscape ecological concepts into planning in current research

##### Empirical papers

Most of the 52 empirical papers in this cohort address urban planning (20 papers) and conservation planning (15), followed by land use planning and landscape planning (both with 8 papers), and landscape restoration (3). Eight papers refer to other types of planning, including strategic environmental assessment and community-based landscape management. Most papers refer to planning at the landscape (28), local (15) and regional level (11).

**Fig. 4:**
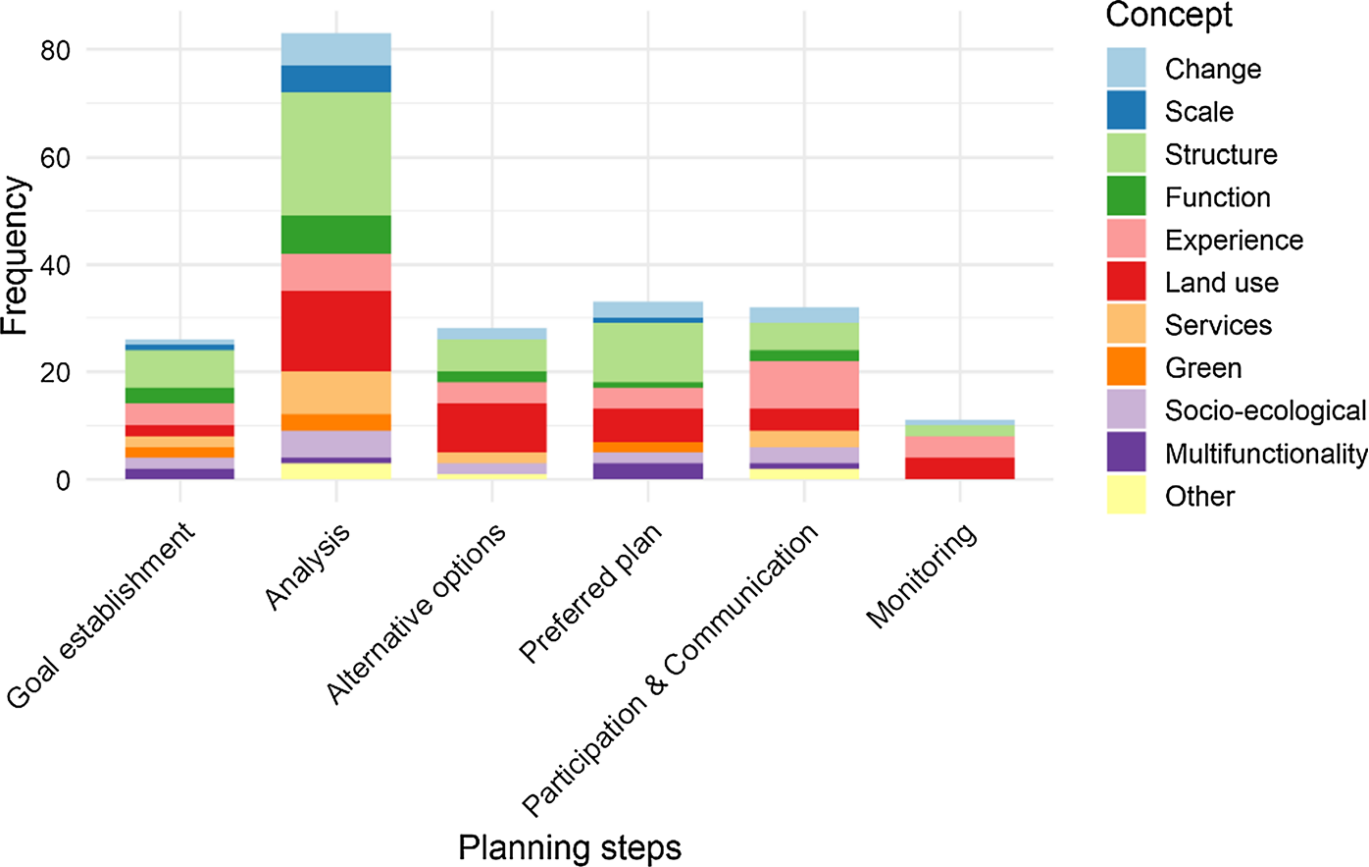
Number of times landscape ecological concepts were addressed in planning steps in the 52 empirical papers analyzed in detail. For the full name of concepts, see Table 2. Concepts were most often addressed in the Landscape analysis step and least in Monitoring

Out of all concepts, only *Structure* is prominent throughout the planning process (Fig. 4, Table D in Supplementary material 3). Also present in all steps are *Land use* and *Landscape as human experience*. The other concepts were only occasionally present and *Holism* and *Stability* were mentioned only once in connection with a planning step (i.e., grouped in category Other in Fig. 4). Most of the 52 papers address landscape ecological concepts in the Analysis step, followed by Preferred plan, Participation and communication, Alternative options, and Goal establishment. Very few papers address landscape ecological concepts in Monitoring. Thus, the concepts are often used for the analysis of the study area, with no deep integration into the entire planning process.

#### Overview papers

In this cohort of 32 papers, eight literature reviews address the integration of landscape ecology into planning. New planning approaches are addressed in reviews on novel ecosystems and socio-ecological resilience by Collier (2015) and on sustainable landscape/landscape sustainability by Zhou et al. (2019). Most reviews focus on integration of specific aspects into planning, i.e., connectivity (Godfree et al. 2017; Costanza and Terando 2019), human perception (Dorning et al. 2017; Mahmoudi and Maller 2018), and urban biodiversity (Norton et al. 2016).

Several papers evaluate plans or projects that have been based on landscape ecological approaches. The focus is on landscape patterns (e.g., Meyer et al. 2015), landscape and ecosystem services (Spyra et al. 2019; van der Sluis et al. 2019), integrated landscape initiatives (Zanzanaini et al. 2017) and urban tree initiatives (Foo and Bebbington 2018). One paper directly addresses the evidence and opportunity for integrating landscape ecology into natural resource planning in public lands of the USA by evaluating the implementation of two plans (Trammell et al. 2018).

Most prominent among the overview contributions are essays and conceptual frameworks. They focus on the potential of planning and management and the role of planners for addressing a range of issues. They relate to landscape and ecosystem services (Musacchio 2018), socio-ecological systems (Fischer 2018), conservation (Gagne et al. 2015), integrated landscape management (Mann et al. 2018), and nature-based solutions (Albert et al. 2019). Two papers of a special issue addressed ecological wisdom (Young 2016; Wang et al. 2016). Most papers, however, provide frameworks and discussions for improving certain aspects of landscape planning and governance: They provide, for example, frameworks for prioritizing green infrastructure (Norton et al. 2015), restoration strategies (Hessburg 2015) and small-scale urban heterogeneity in urban environments (Zhou et al. 2017). Several contributions focus on the planning process for landscape and ecosystem services (e.g., Babí Almenar et al. 2018; Vialatte et al. 2019).

## Discussion

We first reflect on the findings regarding landscape ecological concepts and the frequency of their mentioning (research question 1) and continue with how landscape ecological concepts have been integrated into the six main steps of the planning process (research question 2). We then explore how the additional concepts can support the link between landscape ecology and planning. We also point out limitations of our study and outline potential further research.

### Landscape ecological concepts and their frequency

The most often mentioned concepts include early concepts such as *Change*, *Scale*, *Structure* and *Function*, as well as newer concepts such as *Landscape as human experience*, *Land use* and *Landscape and ecosystem services*. It implies that while the science of landscape ecology is evolving, it is not leaving its roots. Indeed, the distinction between early concepts and additional concepts allows an interpretation of developments over time. Early concepts, particularly *Structure*, *Function*, *Change* and *Scale*, are useful for examining and evaluating landscape patterns and processes and have been used heavily in recent years. Newer concepts emphasize more strongly the use of landscapes for human benefits. This is especially true for concepts such as *Landscape as human experience*, *Land use*, and *Landscape and ecosystem services*. The early concepts focusing on specific systems behavior, i.e., *Chaos theory*, *Hierarchy theory* and *General system theory*, have lost importance and are likely integrated into the new concept *Landscapes as socio-ecological systems*. This change could be interpreted as a transition towards a more applied discipline.

We found additional concepts to be more prevalent than the early concepts in the journals LUP and LO, while the opposite patterns were found in journals CLER and LE. While the differences are rather small, they are in line with the differences in the aims and scopes of the respective journals (see Supplementary material 1). Most importantly, LE and CLER explicitly focus on landscape structure and function or change, while LO and LUP focus on landscapes as human experience.

### Landscape ecological concepts in the steps of the planning process

Surprisingly, out of almost two thousand publications in the four key journals in landscape ecology and landscape planning, only a small number was found promising for analyzing the integration of landscape ecological concepts into landscape planning (52 empirical and 32 overview papers). Many more publications of course recommended in a general statement that their findings may improve planning. These papers provide, for example, novel insights in human–environment interactions and propose new methods to describe and assess landscapes. Many also address landscape ecological concepts. However, a clear link from the concepts to planning, and moreover to planning steps remains the exception.

The inventory and analysis of the biophysical and socioeconomic landscape patterns and processes provide an understanding of how the landscape works (Steiner 2008; Steinitz 2012). This research lends itself to scientific approaches. It is therefore not surprising that we found that most papers addressed landscape ecology concepts in the Analysis step. In contrast, few papers clearly addressed the Preferred plan step, and even when they did, they recommended very generic actions. Notable exceptions are, for example, referring to the design of greenbelts (Siedentop et al. 2016), and the proposal for patches for restoration and protection along preferred routes of movement to build ecological corridors (Babí Almenar et al. 2019). The limited number of papers contributing to the step Monitoring may be because the field of planning evaluation is still evolving (Grădinaru et al. 2020).

In our sample, only few papers connect landscape ecology concepts with all steps of the planning process. We interpret this finding twofold. First, this might be a consequence of the publication tradition: word limits for journal articles make it difficult to address all steps in sufficient detail. Secondly, and perhaps more importantly, the focus on only one or a few planning steps probably reflects a disciplinary division. Landscape ecology scientists might have a limited understanding of the planning process. As the Analysis step fits their experience the best, the link to other steps is done at a more general level.

To overcome the limited integration of landscape ecology concepts in all steps of the planning process, more dialogues between the disciplines are needed. For example, dialogue could be established through conference co-production with landscape ecologists and planners. For the research community, making use of all the publication options (e.g., supplementary material, data in brief, interactive data visualizations) could be a way of describing research on all steps of the planning process in a rigorous manner.

### How landscape ecological concepts can provide a link to planning

Due to its characteristics, each landscape ecological concept offers unique opportunities to link landscape ecological knowledge with planning. The potential use of the early concepts in planning was already explored by Hersperger (1994). Since then, *Structure*, *Function* and *Change* have become key concepts in landscape ecology, and systematic landscape analysis guided by these concepts supports the planning and design of patterns, processes and human–environment interactions. Landscape *Classification* often forms the basis for landscape analysis of this kind. The concept of *Scale* supports analysis in hierarchical systems and is therefore ideally suited to support planning at multiple administrative scales, from neighborhoods to nations. The public often perceives landscapes as holistic entities and therefore *Holism* can be an important aspect in participatory landscape processes. Early theoretical concepts such as *Systems* theory, *Hierarchy* and *Stability* seem to offer less direct links to today's landscape planning. Below, the possible links of the additional concepts to planning are explained in more detail.

#### Landscapes as social ecological systems

An understanding of landscapes as social ecological systems can facilitate the development of integrated models that conceptualize landscapes as nested sets of co-evolving social and natural subsystems connected through feedbacks, time lags, and cross-scale interactions. These models can be used to assess the effects of policies on dynamically linked social and ecological components of the landscape system (Miyasaka et al. 2017). Such models may lead to holistic approaches to manage forest landscape (Fischer 2018) or to resolve land use conflict (Karimi and Hockings 2018).

#### Landscape resilience

To efficiently plan intact natural systems as well as heavily modified landscapes, it is essential to understand how landscapes might react to impacts and challenges. Planning activities based on the *Landscape resilience* concept can help to improve the chances of rapid and effective response to a range of impacts, including extreme events and catastrophes (Ahern 2013; Beller et al. 2018). The *Landscape resilience* concept*,* as well as the *Green infrastructure* concept*,* are thus suited to support planning for climate change mitigation and adaptation.

#### Landscape and ecosystem services

A structured assessment of *Landscape and ecosystem services* supports the design of broadly accepted plans that ensure the optimal provision of multiple services to humans. Furthermore, landscape and ecosystem services have been proposed as a unifying common ground where scientists from various disciplines can cooperate in producing a common knowledge base that can be integrated into multifunctional, actor-led landscape development (Termorshuizen and Opdam 2009).

#### Green infrastructure

The concept of *Green infrastructure* supports the integration of multifunctionality and connectivity into planning. Conceived as a network with patches and corridors, this landscape ecological concept is easily integrated into landscape and spatial planning. Recent research on how users perceive green spaces and which green spaces users prefer has the potential to improve planning for quality of life and health, especially for urban residents (Mahmoudi Farahani et al. 2018). The concept of *Green infrastructure* is well suited to guide the development of planning options and specifically, to support planning for climate change mitigation and adaptation.

#### Multifunctionality

For planning and policy, multifunctionality paves the way for integration of ecological concerns into multiple policy domains, such as climate change through green infrastructure or agricultural policy, illustrated by Common Agricultural Policy in Europe and the Land Stewardship project in Australia (Cocklin et al. 2006). In urban settings, *Multifunctionality* can be used to plan the urban fringe or shift away from mono-functional uses. Its delivery entails integrated planning approaches such as participatory planning (Selman 2009).

#### Land use

The concept is at the heart of land-use and landscape planning. A landscape ecological perspective on land use is expected to provide detailed knowledge on land-use systems and land-use intensity as well as on the management options for sustainable land use. Furthermore, a focus on land use stresses how global environmental change results in severe impacts on biodiversity, and ecosystem integrity and landscape and ecosystem services (Verburg et al. 2015).

#### Landscape as human experience

Participatory landscape planning is closely linked with participants’ landscape experience. Thus, assessments of human landscape experience and landscape perception greatly support landscape planning and design (Downes et al. 2015). The concept *Landscape as human experience* is well suited to represent the heterogeneous expectations towards landscape planning.

Hersperger (1994) suggested that there were only a few applications of landscape ecology concepts into planning of urbanized areas. However, in our sample of recently published research, we found many papers that integrate landscape ecology concepts into urban planning showing that the number of applications has increased and diversified over time. These studies particularly rely on concepts such as *Landscape and ecosystem services*, *Green infrastructure*, *Landscape as human perception* and address planning steps such as analyses, participation and communication. In the same publication, it was furthermore suggested that landscape ecological planning in rural and natural areas mainly focus on conservation planning. We observe that conservation planning continues to be a frequent topic, and we came across many papers that address landscape structure as an important concept for conservation planning, and specifically focus on enhancing landscape connectivity in protected areas.

### Limitations of the analysis

Our findings show that there is limited integration of landscape ecology and planning. A certain bias in the findings could be due to the data in our sample. We focused on the period 2015–2019 in four key journals in the field of landscape ecology and landscape planning to conduct our analysis. While these four journals provide insights into the state-of-the-art research in the field with a broad range of cultural and language regions and easy accessibility, applied research might be underrepresented in our sample. Further research may consider to include other journals (e.g., on landscape architecture, planning practice) or to conduct an analysis on landscape projects.

Furthermore, the assessment on integration of the concepts into planning showed that articles often address this aspect in a general manner. As we collected information on explicit integration into the planning steps, a less conservative approach than ours could lead to different results. Regular planning and project evaluation studies could be useful to observe how effectively landscape ecological concepts have been integrated into planning (see e.g., Hersperger et al. 2020).

### Future research

To overcome the weak integration of landscape ecological concepts into the planning process shown in this research, we propose the following measures. More funding could be provided to research on translating disciplinary landscape ecological research into concepts that can be used in planning. Setting up landscape monitoring systems could encourage both planners and researchers to develop the theoretical aspects related to the Monitoring step. Case studies of landscape ecological planning and the developments of tools to evaluate and monitor the planning activities would be good as a start to promote this dialogue between theory and practice. Journals could open up to publishing more articles on science-practice interactions. For example, formats such as notes or policy briefs could be a way to encourage involvement of landscape ecology scientists in landscape planning. Furthermore, journals could be more rigorous in respect to application of research in planning. Sentences such as “findings can be useful for practice”, which we often encountered in our review, are too general to provide a thorough background for planning practice.

## Conclusions

As an interdisciplinary scientific field, landscape ecology has great potential to inform planning through key concepts of landscape ecology that have been used in the development of the field. Hersperger’s article in 1994 expressed the hope to use the then developing theories and concepts of landscape ecology to change the traditional human-centered environmental planning approach towards a true synthesis of people and nature. After 26 years, responding to the call of the early article of Hersperger (1994), this paper conducted a critical review of the recent development of landscape ecological concepts in planning. It is set to identify the major landscape ecological concepts that have been used frequently by the scientific community in recent years, to explore the causes for their wide usages, and to understand how they may be integrated into different steps of the planning process. To identify the key concepts, we analyzed a total of 1918 empirical and overview papers that have been published in four key academic journals in the field of landscape ecology and landscape planning from 2015 to 2019. To examine the integration of key concepts into planning, we further identified 84 papers from our 1918 paper sample and used them to evaluate how each concept has been integrated into each planning step. Our main findings are the following.

First, while some of the concepts emerged in the early 1990s have remained popular, additional concepts have risen to be frequently used in recent years. Out of the eight promising concepts at the beginning of the 1990s, four have remained pervasive in recent publications, namely *Structure*, *Function*, *Change* and *Scale*. Meanwhile, three additional concepts, i.e., *Landscape as human experience*, *Land use* and *Landscape and ecosystem services*, are widely used in today`s publications, followed by *Green infrastructure* and *Landscape resilience*. While the early concepts leading in usage have been used to examine and evaluate patterns and processes of landscapes, newer concepts emphasize more the use of landscapes for human benefits.

Second, our analysis shows that landscape ecological concepts have not achieved deep integration into the planning process. Out of six planning steps, landscape ecological concepts have been often used in the Analysis and rarely in Goal establishment and Monitoring. Out of all 13 major concepts, *Structure* is mentioned the most as part of the planning process, followed by *Land use*, and *Landscape as human experience*.

The limited number of publications on connecting landscape ecology concepts with all steps of the planning process implied not only a disciplinary division between the fields of landscape ecology and planning but also the current limitation of publication tradition of academic journals. More dialogues between the disciplines are to be encouraged and more publication options can be explored. We emphasized that landscape ecological concepts have great potential to support the planning process, as illustrated by a variety of examples found in the literature. Future studies may include planning-practice oriented journals and landscape projects to more broadly assess the integration of concepts into all key steps of the planning process.

## Supplementary Information

Below is the link to the electronic supplementary material.

(DOCX 59 kb)
