# Supplementary material for: Landscape ecological concepts in planning: review of recent developments
Source: Landsc Ecol. 2021 Jan 28;36(8):2329–45. doi: 10.1007/s10980-021-01193-y (PMC8549942; doi:10.1007/s10980-021-01193-y)
Supplement: Supplementary file 1 — (DOCX 59 kb) [file 10980_2021_1193_MOESM1_ESM.docx]

Supplementary Material

1. ***Database***

Four key journals in the field of landscape ecology were selected to conduct the analysis, respectively Landscape Ecology (LE), Landscape Online (LO), Current Landscape Ecology Reports (CLER), and Landscape and Urban Planning (LUP). The journal selection was based on 1) the relevance for landscape ecology science and 2) the clear linkages between landscape science into planning, based on the journals’ aims and scope descriptions.

According to aims and scopes, CLER focuses on landscape structure and change, ecosystem services and the clear linkage with planning; LE focuses on landscape structure and function, landscape as socio-ecological system, and applied research questions concerning the conservation and landscape planning (between others); LUP focuses on landscape changes, landscapes as human experience, and the premise that landscape science linked to planning and design can provide mutually supportive outcomes for people and nature; LO focuses on landscapes as human experience, landscape as socio-ecological systems, and research questions on landscape theory, system approaches, and conceptual models of landscapes.

Table A. Landscape ecological concepts and variations of terms used for keyword search.

| **Concept** | **Variations of terms** |
| --- | --- |
| Structure, function, change | structure, structures, function, change, changes |
| Stability | stability |
| Chaos Theory | chaos theory |
| Scale | scale, scales |
| Hierarchy Theory | hierarchy theory |
| General Systems Theory | general systems theory |
| Holism | holism, holistic |
| Classification of landscape types | classification of landscape types, classification of landscape, landscape classification, landscape classes. |
| Green and blue infrastructure | green infrastructure(s), green space(s), blue infrastructure(s), blue space(s) |
| Land use | land use(s), land-use(s), naturalness, land use intensity(s), land-use intensity(s) |
| Landscape and ecosystem services | ecosystem service(s), landscape service(s), benefits, landscape character(s), amenities, landscape value(s), recreation, landscape identity(s), landscape conservation(s), landscape disservice(s), ecosystem disservice(s) |
| Landscape as human experience | experience(s), perception(s), landscape meaning(s), perceived landscape(s), soundscape(s), aesthetic(s), appearance(s), visual character(s), landscape preference(s), scenic beauty |
| Landscapes as socio-ecological systems | human-environmental, social-ecological, sustainable landscape(s) |
| Multifunctionality | multifunctionality(s), multifunctional(s) |
| Landscape resilience | resilience(s), resilient(s), integrity(s), resistance(s) |

1. ***Integration of landscape ecological concepts into planning***

By “integration into planning” we refer to: (1) in connection with which topic, (2) to enhance which benefit of planning or to address which issue/ limitation, (3) to be used during which planning step.

The title, abstract and keywords of the papers included in our sample (n=1918 articles) were screened to identify papers which might show how landscape ecological concepts are integrated into planning. This resulted in 131 articles. The 131 papers were assessed for eligibility for the analysis by full-reading them. Papers were excluded if the link to planning was too weak (e.g. one sentence or paragraph addressing planning in a general manner) or if they focus on sectoral management (e.g. forestry, agricultural land management, energy planning). We kept 52 empirical papers and 32 overview papers (Table C, Appendix). The overview papers include frameworks and essays and a research agenda (n=16), evaluations of plans and projects (n=9) and reviews of scientific papers (n=7).

Full reading of the empirical papers (n=52) allowed us to evaluate how landscape ecology concepts have been integrated into planning steps. Based on Steiner (2008), Steinitz (2012) and Botequillha Leitao and Ahern (2002), we identify the following steps: *Goal establishment* (Problem identification and define what is to be achieved); *Analysis* (Biophysical and socioeconomic processes: description and assessment); *Alternative options* (Development of scenarios and assessment of their impact); *Preferred plan* (Decision on a plan chosen for implementation); Participation and communication (possibly all stakeholders and throughout the process); *Monitoring* (Changes in the landscape as well as implementation process).

To systematically collect the data, we used a protocol which addressed the following questions: a) which type of planning is addressed by the paper?, b) to which planning level does the paper refer to?, c) which concepts are integrated in any of the planning steps described above?. The relevant insights on the integration of landscape ecological concepts to planning from the papers on frameworks and essays, evaluations of plans and projects, and the reviews of scientific papers were synthesized after careful reading. To ensure the systematic interpretation when reviewing the articles, we adopted the following four-step procedure. First, two of the authors applied the protocol on a small sample of both empirical and overview articles. Second, based on the results of the interpretation, the authors calibrated the protocol to assure that interpretation follows the same line. Third, article reading was split among three of the authors. Finally, after all papers were read, the three authors discussed the results and agreed on interpretation. Particular attention was payed to papers which were unclear, for example on integration of landscape ecology concepts into the planning steps.

1. ***Results***

Table B. Frequency in the use of each early concept by the journals (data accompanying Figure 2a)

|  | **Journal** | | | |
| --- | --- | --- | --- | --- |
| **Concept** | CLER | LE | LO | LUP |
| Change | 617 | 7138 | 241 | 7648 |
| Scale | 614 | 5538 | 119 | 3430 |
| Structure | 388 | 2651 | 154 | 2488 |
| Function | 90 | 1190 | 27 | 1257 |
| Stability | 14 | B181 | 3 | 147 |
| Holism | 6 | 65 | 23 | 204 |
| Classification | 0 | 22 | 6 | 6 |
| Hierarchy | 1 | 4 | 1 | 2 |
| System Theory | 0 | 1 | 0 | 1 |

Table C Frequency in the use of each early concept by the journals (data accompanying Figure 2b)

|  | **Journal** | | | |
| --- | --- | --- | --- | --- |
| **Concept** | CLER | LE | LO | LUP |
| Experience | 182 | 946 | 278 | 6493 |
| Land use | 268 | 2776 | 229 | 5436 |
| Services | 189 | 2178 | 522 | 4057 |
| Green | 54 | 202 | 53 | 3979 |
| Resilience | 59 | 2069 | 22 | 1088 |
| Socio-ecological | 21 | 143 | 23 | 246 |
| Multifunctionality | 5 | 225 | 30 | 164 |

Table D. Number of times landscape ecological concepts were addressed in planning steps in the 52 empirical papers analyzed in detail (data accompanying Figure 4)

| **Concept** | **Planning step** | | | | | | |
| --- | --- | --- | --- | --- | --- | --- | --- |
|  | **Goal establishment** | **Analysis** | **Alternative options** | **Preferred plan** | **Participation & Communication** | **Monitoring** |  |
| Change | 1 | 6 | 2 | 3 | 3 | 1 |  |
| Scale | 1 | 5 | 0 | 1 | 0 | 0 |  |
| Structure | 7 | 23 | 6 | 11 | 5 | 2 |  |
| Function | 3 | 7 | 2 | 1 | 2 | 0 |  |
| Experience | 4 | 7 | 4 | 4 | 9 | 4 |  |
| Land use | 2 | 15 | 9 | 6 | 4 | 4 |  |
| Services | 2 | 8 | 2 | 0 | 3 | 0 |  |
| Green | 2 | 3 | 0 | 2 | 0 | 0 |  |
| Socio-ecological | 2 | 5 | 2 | 2 | 3 | 0 |  |
| Multifunctionality | 2 | 1 | 0 | 3 | 1 | 0 |  |
| Other | 0 | 3 | 1 | 0 | 2 | 0 |  |
| **Total** | **26** | **83** | **28** | **33** | **32** | **11** |  |

1. ***List of publications analyzed in detail***

Table E. Publications analyzed in detail for assessing the integration of landscape ecological concepts into planning. Journal abbreviations: Landscape Ecology (LE), Landscape Online (LO), Current Landscape Ecology Reports (CLER), and Landscape and Urban Planning (LUP).

| **Publications analyzed in detail** | **Journal** | **Year** | **Type** |
| --- | --- | --- | --- |
| Albano CM (2015) Identification of geophysically diverse locations that may facilitate species’ persistence and adaptation to climate change in the southwestern United States. Landsc Ecol 30:1023-1037.<https://doi.org/10.1007/s10980-015-0167-7> | LE | 2015 | Empirical |
| Albert C, Schröter B, Haase D, Brillinger M, Henze J, Herrmann S, Gottwald S, Guerrero P, Nicolas C, Matzdorf B (2019) Addressing societal challenges through nature-based solutions: How can landscape planning and governance research contribute? Landsc Urban Plan 182:12-21.<https://doi.org/10.1016/j.landurbplan.2018.10.003> | LUP | 2019 | Overview |
| Avon C, Bergès L (2016) Prioritization of habitat patches for landscape connectivity conservation differs between least-cost and resistance distances. Landsc Ecol 31:1551-1565.<https://doi.org/10.1007/s10980-015-0336-8> | LE | 2016 | Empirical |
| Babí Almenar J, Bolowich A, Elliot T, Geneletti D, Sonnemann G, Rugani B (2019) Assessing habitat loss, fragmentation and ecological connectivity in Luxembourg to support spatial planning. Landsc Urban Plan 189:335-351.<https://doi.org/10.1016/j.landurbplan.2019.05.004> | LUP | 2019 | Empirical |
| Babí Almenar J, Rugani B, Geneletti D, Brewer T (2018) Integration of ecosystem services into a conceptual spatial planning framework based on a landscape ecology perspective. Landsc Ecol 33:2047-2059.<https://doi.org/10.1007/s10980-018-0727-8> | LE | 2018 | Overview |
| Bacher M, Walde JF, Pecher C, Tasser E, Tappeiner U (2016) Are interest groups different in the factors determining landscape preferences? Landsc Online 47:1-18.<https://doi.org/10.3097/lo.201647> | LO | 2016 | Empirical |
| Bagstad KJ, Semmens DJ, Ancona ZH, Sherrouse BC (2017) Evaluating alternative methods for biophysical and cultural ecosystem services hotspot mapping in natural resource planning. Landsc Ecol 32:77-97.<https://doi.org/10.1007/s10980-016-0430-6> | LE | 2017 | Empirical |
| Bakker M, Alam SJ, van Dijk J, Rounsevell M, Spek T, van den Brink A (2015) The feasibility of implementing an ecological network in The Netherlands under conditions of global change. Landsc Ecol 30:791-804.<https://doi.org/10.1007/s10980-014-0145-5> | LE | 2015 | Evaluation |
| Bartlett D, Gomez-Martin E, Milliken S, Parmer D (2017) Introducing landscape character assessment and the ecosystem service approach to India: A case study. Landsc Urban Plan 167:257-266.<https://doi.org/10.1016/j.landurbplan.2017.06.013> | LUP | 2017 | Empirical |
| Behrman KD, Juenger TE, Kiniry JR, Keitt TH (2015) Spatial land use trade-offs for maintenance of biodiversity, biofuel, and agriculture. Landsc Ecol 30:1987-1999.<https://doi.org/10.1007/s10980-015-0225-1> | LE | 2015 | Empirical |
| Belda-Carrasco R, Iranzo-García E, Pascual-Aguilar JA (2019) Landscape Dynamics in Mediterranean Coastal Areas: Castelló de la Plana in the Last Hundred Years. Landsc Online 69:1-15.<https://doi.org/10.3097/lo.201969> | LO | 2019 | Empirical |
| Bell KP, Markowski-Lindsay M, Catanzaro P, Leahy J (2019) Family-forest owner decisions, landscape context, and landscape change. Landsc Urban Plan 188:118-131.<https://doi.org/10.1016/j.landurbplan.2018.08.023> | LUP | 2019 | Empirical |
| Beller EE, Downs PW, Grossinger RM, Orr BK, Salomon MN (2016) From past patterns to future potential: using historical ecology to inform river restoration on an intermittent California river. Landsc Ecol 31:581-600.<https://doi.org/10.1007/s10980-015-0264-7> | LE | 2016 | Empirical |
| Bergillos RJ, Ortega-Sánchez M (2017) Assessing and mitigating the landscape effects of river damming on the Guadalfeo River delta, southern Spain. Landsc Urban Plan 165:117-129.<https://doi.org/10.1016/j.landurbplan.2017.05.002> | LUP | 2017 | Empirical |
| Bobiec A, Podlaski R, Ortyl B, Korol M, Havryliuk S, Öllerer K, Ziobro JM, Pilch K, Dychkevych V, Dudek T, Mázsa K, Varga A, Angelstam P (2019) Top-down segregated policies undermine the maintenance of traditional wooded landscapes: Evidence from oaks at the European Union’s eastern border. Landsc Urban Plan 189:247-259.<https://doi.org/10.1016/j.landurbplan.2019.04.026> | LUP | 2019 | Empirical |
| Carlsson J, Lidestav G, Bjärstig T, Svensson J, Nordström E-M (2017) Opportunities for Integrated Landscape Planning – the Broker, the Arena, the Tool. Landsc Online 55:1-20.<https://doi.org/10.3097/lo.201755> | LO | 2017 | Empirical |
| Celio E, Ott M, Sirén E, Grêt-Regamey A (2015) A prototypical tool for normative landscape scenario development and the analysis of actors’ policy preferences. Landsc Urban Plan 137:40-53.<https://doi.org/10.1016/j.landurbplan.2014.12.013> | LUP | 2015 | Empirical |
| Cimon-Morin J, Darveau M, Poulin M (2016) Consequences of delaying conservation of ecosystem services in remote landscapes prone to natural resource exploitation. Landsc Ecol 31:825-842.<https://doi.org/10.1007/s10980-015-0291-4> | LE | 2016 | Empirical |
| Clauzel C, Jeliazkov A, Mimet A (2018) Coupling a landscape-based approach and graph theory to maximize multispecific connectivity in bird communities. Landsc Urban Plan 179:1-16.<https://doi.org/10.1016/j.landurbplan.2018.07.002> | LUP | 2018 | Empirical |
| Colléony A, Prévot A-C, Saint Jalme M, Clayton S (2017) What kind of landscape management can counteract the extinction of experience? Landsc Urban Plan 159:23-31.<https://doi.org/10.1016/j.landurbplan.2016.11.010> | LUP | 2017 | Empirical |
| Collier MJ (2015) Novel ecosystems and social-ecological resilience. Landsc Ecol 30:1363-1369.<https://doi.org/10.1007/s10980-015-0243-z> | LE | 2015 | Review |
| Costanza JK, Terando AJ (2019) Landscape Connectivity Planning for Adaptation to Future Climate and Land-Use Change. Curr Landsc Ecol Rep 4:1-13.<https://doi.org/10.1007/s40823-019-0035-2> | CLER | 2019 | Review |
| Dorning MA, Van Berkel DB, Semmens DJ (2017) Integrating Spatially Explicit Representations of Landscape Perceptions into Land Change Research. Curr Landsc Ecol Rep 2:73-88.<https://doi.org/10.1007/s40823-017-0025-1> | CLER | 2017 | Review |
| Dupont L, Antrop M, Van Eetvelde V (2015) Does landscape related expertise influence the visual perception of landscape photographs? Implications for participatory landscape planning and management. Landsc Urban Plan 141:68-77.<https://doi.org/10.1016/j.landurbplan.2015.05.003> | LUP | 2015 | Empirical |
| Dupont L, Ooms K, Antrop M, Van Eetvelde V (2016) Comparing saliency maps and eye-tracking focus maps: The potential use in visual impact assessment based on landscape photographs. Landsc Urban Plan 148:17-26.<https://doi.org/10.1016/j.landurbplan.2015.12.007> | LUP | 2016 | Empirical |
| Farinha-Marques P, Fernandes C, Guilherme F, Lameiras JM, Alves P, Bunce RGH (2017) Urban Habitats Biodiversity Assessment (UrHBA): a standardized procedure for recording biodiversity and its spatial distribution in urban environments. Landsc Ecol 32:1753-1770.<https://doi.org/10.1007/s10980-017-0554-3> | LE | 2017 | Empirical |
| Fischer AP (2018) Forest landscapes as social-ecological systems and implications for management. Landsc Urban Plan 177:138-147.<https://doi.org/10.1016/j.landurbplan.2018.05.001> | LUP | 2018 | Overview |
| Fischer AP, Klooster A, Cirhigiri L (2019) Cross-boundary cooperation for landscape management: Collective action and social exchange among individual private forest landowners. Landsc Urban Plan 188:151-162.<https://doi.org/10.1016/j.landurbplan.2018.02.004> | LUP | 2019 | Empirical |
| Fischer AP, Vance-Borland K, Jasny L, Grimm KE, Charnley S (2016) A network approach to assessing social capacity for landscape planning: The case of fire-prone forests in Oregon, USA. Landsc Urban Plan 147:18-27.<https://doi.org/10.1016/j.landurbplan.2015.10.006> | LUP | 2016 | Empirical |
| Foo K, McCarthy J, Bebbington A (2018) Activating landscape ecology: a governance framework for design-in-science. Landsc Ecol 33:675-689.<https://doi.org/10.1007/s10980-018-0630-3> | LE | 2018 | Evaluation |
| Foster E, Love J, Rader R, Reid N, Drielsma MJ (2017) Integrating a generic focal species, metapopulation capacity, and connectivity to identify opportunities to link fragmented habitat. Landsc Ecol 32:1837-1847.<https://doi.org/10.1007/s10980-017-0547-2> | LE | 2017 | Empirical |
| Gagné SA, Eigenbrod F, Bert DG, Cunnington GM, Olson LT, Smith AC, Fahrig L (2015) A simple landscape design framework for biodiversity conservation. Landsc Urban Plan 136:13-27.<https://doi.org/10.1016/j.landurbplan.2014.11.006> | LUP | 2015 | Overview |
| Garcia-Martin M, Fagerholm N, Bieling C, Gounaridis D, Kizos T, Printsmann A, Müller M, Lieskovský J, Plieninger T (2017) Participatory mapping of landscape values in a Pan-European perspective. Landsc Ecol 32:2133-2150.<https://doi.org/10.1007/s10980-017-0531-x> | LE | 2017 | Empirical |
| Geneletti D, La Rosa D, Spyra M, Cortinovis C (2017) A review of approaches and challenges for sustainable planning in urban peripheries. Landsc Urban Plan 165:231-243.<https://doi.org/10.1016/j.landurbplan.2017.01.013> | LUP | 2017 | Review |
| Glenn DT, Endter-Wada J, Kjelgren R, Neale CMU (2015) Tools for evaluating and monitoring effectiveness of urban landscape water conservation interventions and programs. Landsc Urban Plan 139:82-93.<https://doi.org/10.1016/j.landurbplan.2015.03.002> | LUP | 2015 | Empirical |
| Godfree R, Firn J, Johnson S, Knerr N, Stol J, Doerr V (2017) Why non-native grasses pose a critical emerging threat to biodiversity conservation, habitat connectivity and agricultural production in multifunctional rural landscapes. Landsc Ecol.<https://doi.org/10.1007/s10980-017-0516-9> | LE | 2017 | Evaluation |
| Groot JCJ, Yalew SG, Rossing WAH (2018) Exploring ecosystem services trade-offs in agricultural landscapes with a multi-objective programming approach. Landsc Urban Plan 172:29-36.<https://doi.org/10.1016/j.landurbplan.2017.12.008> | LUP | 2018 | Overview |
| Guo T, Smith JW, Moore RL, Schultz CL (2017) Integrating off-site visitor education into landscape conservation and management: An examination of timing of educational messaging and compliance with low-impact hiking recommendations. Landsc Urban Plan 164:25-36.<https://doi.org/10.1016/j.landurbplan.2017.03.013> | LUP | 2017 | Empirical |
| Hadavi S, Kaplan R, Hunter MCR (2015) Environmental affordances: A practical approach for design of nearby outdoor settings in urban residential areas. Landsc Urban Plan 134:19-32.<https://doi.org/10.1016/j.landurbplan.2014.10.001> | LUP | 2015 | Empirical |
| Haines AL, Thompson AW, McFarlane D, Sharp AK (2019) Local policy and landowner attitudes: A case study of forest fragmentation. Landsc Urban Plan 188:97-109.<https://doi.org/10.1016/j.landurbplan.2018.08.026> | LUP | 2019 | Empirical |
| Hegland SJ, Hamre LN (2018) Scale-dependent effects of landscape composition and configuration on deer-vehicle collisions and their relevance to mitigation and planning options. Landsc Urban Plan 169:178-184.<https://doi.org/10.1016/j.landurbplan.2017.09.009> | LUP | 2018 | Empirical |
| Hernández-Morcillo M, Bieling C, Bürgi M, Lieskovský J, Palang H, Printsmann A, Schulp CJE, Verburg PH, Plieninger T (2017) Priority questions for the science, policy and practice of cultural landscapes in Europe. Landsc Ecol 32:2083-2096.<https://doi.org/10.1007/s10980-017-0524-9> | LE | 2017 | Overview |
| Herrero-Jáuregui C, Arnaiz-Schmitz C, Herrera L, Smart SM, Montes C, Pineda FD, Schmitz MF (2019) Aligning landscape structure with ecosystem services along an urban–rural gradient. Trade-offs and transitions towards cultural services. Landsc Ecol 34:1525-1545.<https://doi.org/10.1007/s10980-018-0756-3> | LE | 2019 | Empirical |
| Hessburg PF, Churchill DJ, Larson AJ, Haugo RD, Miller C, Spies TA, North MP, Povak NA, Belote RT, Singleton PH, Gaines WL, Keane RE, Aplet GH, Stephens SL, Morgan P, Bisson PA, Rieman BE, Salter RB, Reeves GH (2015) Restoring fire-prone Inland Pacific landscapes: seven core principles. Landsc Ecol 30:1805-1835.<https://doi.org/10.1007/s10980-015-0218-0> | LE | 2015 | Overview |
| Hoyle H, Hitchmough J, Jorgensen A (2017) Attractive, climate-adapted and sustainable? Public perception of non-native planting in the designed urban landscape. Landsc Urban Plan 164:49-63.<https://doi.org/10.1016/j.landurbplan.2017.03.009> | LUP | 2017 | Empirical |
| Hu H, Fu B, Lü Y, Zheng Z (2015) SAORES: a spatially explicit assessment and optimization tool for regional ecosystem services. Landsc Ecol 30:547-560.<https://doi.org/10.1007/s10980-014-0126-8> | LE | 2015 | Empirical |
| Karimi A, Hockings M (2018) A social-ecological approach to land-use conflict to inform regional and conservation planning and management. Landsc Ecol 33:691-710.<https://doi.org/10.1007/s10980-018-0636-x> | LE | 2018 | Empirical |
| Kashian DM, Sosin JR, Huber PW, Tucker MM, Dombrowski J (2017) A neutral modeling approach for designing spatially heterogeneous jack pine plantations in northern Lower Michigan, USA. Landsc Ecol 32:1117-1131.<https://doi.org/10.1007/s10980-017-0514-y> | LE | 2017 | Empirical |
| Kim G, Kang W, Park CR, Lee D (2018) Factors of spatial distribution of Korean village groves and relevance to landscape conservation. Landsc Urban Plan 176:30-37.<https://doi.org/10.1016/j.landurbplan.2018.03.015> | LUP | 2018 | Empirical |
| Kowarik I (2019) The “Green Belt Berlin”: Establishing a greenway where the Berlin Wall once stood by integrating ecological, social and cultural approaches. Landsc Urban Plan 184:12-22.<https://doi.org/10.1016/j.landurbplan.2018.12.008> | LUP | 2019 | Empirical |
| Kukkala AS, Moilanen A (2017) Ecosystem services and connectivity in spatial conservation prioritization. Landsc Ecol 32:5-14.<https://doi.org/10.1007/s10980-016-0446-y> | LE | 2017 | Overview |
| Le Roux M, Redon M, Archaux F, Long J, Vincent S, Luque S (2017) Conservation planning with spatially explicit models: a case for horseshoe bats in complex mountain landscapes. Landsc Ecol 32:1005-1021.<https://doi.org/10.1007/s10980-017-0505-z> | LE | 2017 | Empirical |
| Lechner AM, Brown G, Raymond CM (2015) Modeling the impact of future development and public conservation orientation on landscape connectivity for conservation planning. Landsc Ecol 30:699-713.<https://doi.org/10.1007/s10980-015-0153-0> | LE | 2015 | Empirical |
| Lechner AM, Sprod D, Carter O, Lefroy EC (2017) Characterising landscape connectivity for conservation planning using a dispersal guild approach. Landsc Ecol 32:99-113.<https://doi.org/10.1007/s10980-016-0431-5> | LE | 2017 | Empirical |
| Ma B, Tian G, Kong L, Liu X (2018) How China’s linked urban–rural construction land policy impacts rural landscape patterns: a simulation study in Tianjin, China. Landsc Ecol 33:1417-1434.<https://doi.org/10.1007/s10980-018-0669-1> | LE | 2018 | Evaluation |
| Magness DR, Sesser AL, Hammond T (2018) Using topographic geodiversity to connect conservation lands in the Central Yukon, Alaska. Landsc Ecol 33:547-556.<https://doi.org/10.1007/s10980-018-0617-0> | LE | 2018 | Empirical |
| Mahmoudi Farahani L, Maller CJ (2018) Perceptions and Preferences of Urban Greenspaces: A Literature Review and Framework for Policy and Practice. Landsc Online 61:1-22.<https://doi.org/10.3097/lo.201861> | LO | 2018 | Overview |
| Mahmoudi Farahani L, Maller C, Phelan K (2018) Private Gardens as Urban Greenspaces: Can They Compensate for Poor Greenspace Access in Lower Socioeconomic Neighbourhoods? Landsc Online 59:1-18.<https://doi.org/10.3097/lo.201859> | LO | 2018 | Empirical |
| Mann C, Garcia-Martin M, Raymond CM, Shaw BJ, Plieninger T (2018) The potential for integrated landscape management to fulfil Europe’s commitments to the Sustainable Development Goals. Landsc Urban Plan 177:75-82.<https://doi.org/10.1016/j.landurbplan.2018.04.017> | LUP | 2018 | Overview |
| McGarigal K, Compton BW, Plunkett EB, DeLuca WV, Grand J, Ene E, Jackson SD (2018) A landscape index of ecological integrity to inform landscape conservation. Landsc Ecol 33:1029-1048.<https://doi.org/10.1007/s10980-018-0653-9> | LE | 2018 | Empirical |
| Meyer SR, Beard K, Cronan CS, Lilieholm RJ (2015) An analysis of spatio-temporal landscape patterns for protected areas in northern New England: 1900–2010. Landsc Ecol 30:1291-1305.<https://doi.org/10.1007/s10980-015-0184-6> | LE | 2015 | Evaluation |
| Musacchio LR (2018) Ecologies as a complement to ecosystem services? Exploring how landscape planners might advance understanding about human–nature relationships in changing landscapes. Landsc Ecol 33:847-860.<https://doi.org/10.1007/s10980-018-0646-8> | LE | 2018 | Overview |
| Norton BA, Coutts AM, Livesley SJ, Harris RJ, Hunter AM, Williams NSG (2015) Planning for cooler cities: A framework to prioritise green infrastructure to mitigate high temperatures in urban landscapes. Landsc Urban Plan 134:127-138.<https://doi.org/10.1016/j.landurbplan.2014.10.018> | LUP | 2015 | Overview |
| Norton BA, Evans KL, Warren PH (2016) Urban Biodiversity and Landscape Ecology: Patterns, Processes and Planning. Curr Landsc Ecol Rep 1:178-192.<https://doi.org/10.1007/s40823-016-0018-5> | CLER | 2016 | Review |
| Oudes D, Stremke S (2018) Spatial transition analysis: Spatially explicit and evidence-based targets for sustainable energy transition at the local and regional scale. Landsc Urban Plan 169:1-11.<https://doi.org/10.1016/j.landurbplan.2017.07.018> | LUP | 2018 | Empirical |
| Pirnat J, Hladnik D (2016) Connectivity as a tool in the prioritization and protection of sub-urban forest patches in landscape conservation planning. Landsc Urban Plan 153:129-139.<https://doi.org/10.1016/j.landurbplan.2016.05.013> | LUP | 2016 | Empirical |
| Prasad AM, Iverson LR, Matthews SN, Peters MP (2016) A multistage decision support framework to guide tree species management under climate change via habitat suitability and colonization models, and a knowledge-based scoring system. Landsc Ecol 31:2187-2204.<https://doi.org/10.1007/s10980-016-0369-7> | LE | 2016 | Empirical |
| Raatikainen K (2018) The Importance of Engaging Local People in Landscape Management – Experiences from an EU Project. Landsc Online 57:1-22.<https://doi.org/10.3097/lo.201857> | LO | 2018 | Empirical |
| Sethi SA, O’Hanley JR, Gerken J, Ashline J, Bradley C (2017) High value of ecological information for river connectivity restoration. Landsc Ecol 32:2327-2336.<https://doi.org/10.1007/s10980-017-0571-2> | LE | 2017 | Empirical |
| Siedentop S, Fina S, Krehl A (2016) Greenbelts in Germany's regional plans—An effective growth management policy? Landsc Urban Plan 145:71-82.<https://doi.org/10.1016/j.landurbplan.2015.09.002> | LUP | 2016 | Evaluation |
| Spyra M, Kleemann J, Cetin NI, Vázquez Navarrete CJ, Albert C, Palacios-Agundez I, Ametzaga-Arregi I, La Rosa D, Rozas-Vásquez D, Adem Esmail B, Picchi P, Geneletti D, König HJ, Koo H, Kopperoinen L, Fürst C (2019) The ecosystem services concept: a new Esperanto to facilitate participatory planning processes? Landsc Ecol 34:1715-1735.<https://doi.org/10.1007/s10980-018-0745-6> | LE | 2019 | Evaluation |
| Tieskens KF, Shaw BJ, Haer T, Schulp CJE, Verburg PH (2017) Cultural landscapes of the future: using agent-based modeling to discuss and develop the use and management of the cultural landscape of South West Devon. Landsc Ecol 32:2113-2132.<https://doi.org/10.1007/s10980-017-0502-2> | LE | 2017 | Empirical |
| Trammell EJ, Carter SK, Haby T, Taylor JJ (2018) Evidence and Opportunities for Integrating Landscape Ecology into Natural Resource Planning across Multiple-Use Landscapes. Curr Landsc Ecol Rep 3:1-11.<https://doi.org/10.1007/s40823-018-0029-5> | CLER | 2018 | Evaluation |
| Van der Sluis T, Pedroli B, Frederiksen P, Kristensen SBP, Busck AG, Pavlis V, Cosor GL (2019) The impact of European landscape transitions on the provision of landscape services: an explorative study using six cases of rural land change. Landsc Ecol 34:307-323.<https://doi.org/10.1007/s10980-018-0765-2> | LE | 2019 | Evaluation |
| Vialatte A, Barnaud C, Blanco J, Ouin A, Choisis J-P, Andrieu E, Sheeren D, Ladet S, Deconchat M, Clément F, Esquerré D, Sirami C (2019) A conceptual framework for the governance of multiple ecosystem services in agricultural landscapes. Landsc Ecol 34:1653-1673.<https://doi.org/10.1007/s10980-019-00829-4> | LE | 2019 | Overview |
| Virah-Sawmy M, Gillson L, Gardner CJ, Anderson A, Clark G, Haberle S (2016) A landscape vulnerability framework for identifying integrated conservation and adaptation pathways to climate change: the case of Madagascar’s spiny forest. Landsc Ecol 31:637-654.<https://doi.org/10.1007/s10980-015-0269-2> | LE | 2016 | Empirical |
| Wang T et al. (2016a) Amur tigers and leopards returning to China: direct evidence and a landscape conservation plan. Landsc Ecol 31:491-503. https://doi.org/10.1007/s10980-015-0278-1 | LE | 2016 | Empirical |
| [Wang X, Palazzo D, Carper M (2016b) Ecological wisdom as an emerging field of scholarly inquiry in urban planning and design. Landsc Urban Plan 155:100-107. https://doi.org/10.1016/j.landurbplan.2016.05.019](https://doi.org/10.1016/j.landurbplan.2016.05.019) | LUP | 2016 | Overview |
| Xun B, Yu D, Wang X (2017) Prioritizing habitat conservation outside protected areas in rapidly urbanizing landscapes: A patch network approach. Landsc Urban Plan 157:532-541.<https://doi.org/10.1016/j.landurbplan.2016.09.013> | LUP | 2017 | Empirical |
| Young RF (2016) Modernity, postmodernity, and ecological wisdom: Toward a new framework for landscape and urban planning. Landsc Urban Plan 155:91-99.<https://doi.org/10.1016/j.landurbplan.2016.04.012> | LUP | 2016 | Overview |
| Zanzanaini C, Trần BT, Singh C, Hart A, Milder J, DeClerck F (2017) Integrated landscape initiatives for agriculture, livelihoods and ecosystem conservation: An assessment of experiences from South and Southeast Asia. Landsc Urban Plan 165:11-21.<https://doi.org/10.1016/j.landurbplan.2017.03.010> | LUP | 2017 | Evaluation |
| Zhang Z, Wang B, Buyantuev A, He X, Gao W, Wang Y, Dawazhaxi, Yang Z (2019) Urban agglomeration of Kunming and Yuxi cities in Yunnan, China: the relative importance of government policy drivers and environmental constraints. Landsc Ecol 34:663-679.<https://doi.org/10.1007/s10980-019-00790-2> | LE | 2019 | Empirical |
| Zhou B-B, Wu J, Anderies JM (2019) Sustainable landscapes and landscape sustainability: A tale of two concepts. Landsc Urban Plan 189:274-284.<https://doi.org/10.1016/j.landurbplan.2019.05.005> | LUP | 2019 | Review |
| Zhou W, Pickett STA, Cadenasso ML (2017) Shifting concepts of urban spatial heterogeneity and their implications for sustainability. Landsc Ecol 32:15-30.<https://doi.org/10.1007/s10980-016-0432-4> | LE | 2017 | Overview |
